# Tau in the Pathophysiology of Parkinson’s Disease

**DOI:** 10.1007/s12031-020-01776-5

**Published:** 2021-01-18

**Authors:** Lina Pan, Lanxia Meng, Mingyang He, Zhentao Zhang

**Affiliations:** Department of Neurology, Renmin Hospital of Wuhan University, Wuhan, 430060 China

**Keywords:** Parkinson’s disease, α-Synuclein, Tau, Pathophysiology

## Abstract

The pathological hallmarks of Parkinson's disease (PD) are the progressive loss of dopaminergic neurons in the substantia nigra and the formation of Lewy bodies (LBs) in remaining neurons. LBs primarily consist of aggregated α-Synuclein (α-Syn). However, accumulating evidence suggests that Tau, which is associated with tauopathies such as Alzheimer’s disease (AD), progressive supranuclear palsy (PSP), and argyrophilic grain disease, is also involved in the pathophysiology of PD. A genome-wide association study (GWAS) identified *MAPT*, the gene encoding the Tau protein, as a risk gene for PD. Autopsy of PD patients also revealed the colocalization of Tau and α-Syn in LBs. Experimental evidence has shown that Tau interacts with α-Syn and influences the pathology of α-Syn in PD. In this review, we discuss the structure and function of Tau and provide a summary of the current evidence supporting Tau’s involvement as either an active or passive element in the pathophysiology of PD, which may provide novel targets for the early diagnosis and treatment of PD.

## Background

Parkinson’s disease (PD), one of the most common neurodegenerative diseases, is currently incurable. The prevalence and incidence of PD in industrialized countries are 1.0% in those over 60 years old and 8 to 18 per 100,000 person-years, respectively. Age is the strongest risk factor for PD (Balestrino and Schapira 2020). With global aging, PD is taking an increasing toll on medical resources, but its etiology remains unclear. Age and male sex are also independent risk factors for PD, and exposure to pesticides and traumatic brain injury increases the risk of PD. In contrast, reduced risk of PD is associated with smoking, caffeine consumption, higher serum urate concentrations, and physical activity (Ascherio and Schwarzschild 2016). In addition to environmental risk factors, genetic factors also play an important role in PD. Approximately 15% of PD patients have a family history, whereas 5–10% have monogenic forms of the disease. At least 23 loci and 19 disease-causing genes and various genetic risk factors have been identified to date (Deng et al. 2018). Variants in genes such as *SNCA* (synuclein alpha, encoding α-Syn), *GBA* (glucosylceramidase beta, encoding GBA protein), *LRRK2* (leucine-rich repeat kinase 2, encoding LRRK2 protein), and *MAPT* (microtubule associated protein, encoding Tau) have been found to increase the risk of PD (Bras and Singleton 2009).

The progressive loss of dopaminergic neurons in the substantia nigra pars compacta (SNpc) and the formation of Lewy bodies (LBs) in the remaining neurons are pathological characteristics of PD (Arima et al. 1999; Kaur et al. 2019). The clinical features of PD manifest as motor as well as nonmotor symptoms. Resting tremor, bradykinesia, rigidity, and postural instability are the primary motor symptoms of PD (Sveinbjornsdottir 2016). Nonmotor symptoms such as depression, constipation, sleep disorders, and dementia can appear earlier and significantly affect quality of life (Chaudhuri et al. 2006). Levodopa is the mainstay treatment to alleviate motor symptoms (Reich and Savitt 2019). In recent years, deep brain stimulation (DBS) has become an optional therapeutic method for patients with advanced PD (Hacker et al. 2020; Krack et al. 2003).

Currently, several issues remain in the field of PD. First, the etiology and pathogenesis of PD remain elusive. Second, the diagnosis of PD relies on its motor symptoms. However, it is difficult to establish clinical acumen to make an early accurate diagnosis, though pathological lesions may appear decades before the onset of clinical symptoms. The establishment of early biological markers can facilitate the early diagnosis of PD. Third, pharmacologic treatment at the late stage of the disease has intractable side effects. Accordingly, there is an urgent need for disease-modifying drugs that can halt or slow the development of PD. To solve these problems, more research on the mechanisms underlying the pathophysiology of PD needs to be conducted.

LBs are mainly composed of aggregated forms of the presynaptic protein α-Synuclein (α-Syn), which is an abundant neuronal protein that localizes predominantly to presynaptic terminals (Kaur et al. 2019; Teravskis et al. 2018). α-Syn aggregates into fibrillary assemblies during the onset of PD, and the aberrant aggregation of α-Syn is considered to contribute to the pathogenesis of PD (Wakabayashi et al. 2013). However, the molecular mechanisms underlying the aggregation of α-Syn remain unknown. In 1999, Arima et al. detected colocalization of α-Syn and Tau, a predominant pathological element in Alzheimer’s disease (AD), in LBs of PD patients (Arima et al. 1999). Later, a genome-wide association study (GWAS) identified *MAPT*, the gene encoding Tau, as a risk gene for PD (Edwards et al. 2010; International Parkinson’s Disease Genomics Consortium et al. 2017). Since then, extensive investigations have been performed to explore the roles of Tau in PD. Researchers have also found a significant association between Tau levels in the cerebrospinal fluid (CSF) and clinical manifestations in PD patients (W. T. Hu et al. 2010). Furthermore, several in vitro and in vivo studies have explored the roles of Tau in PD (Beauchamp 2018; Shi et al. 2016; Singh et al. 2019). These findings indicate that Tau contributes to PD pathology as an underappreciated component and may provide a novel therapeutic target for PD.

## PD Pathophysiology and Diagnosis

Currently, the molecular mechanisms underlying the pathophysiology of PD remain largely unknown. To date, α-Syn accumulation, mitochondrial dysfunction, oxidative stress, and excitotoxicity are thought to play crucial roles in PD pathophysiology (Kaur et al. 2019).

The accumulation of α-Syn progresses predictably throughout the brain, typically known as the Braak stage. The lesions initially begin from the dorsal motor nucleus of the glossopharyngeal and vagal nerves and the anterior olfactory nucleus. They then ascend to the brain stem and finally to the neocortex (Braak et al. 2003). The reasons why α-Syn, an intrinsically disordered protein, misfolds and deposits in the brain are still obscure. The currently accepted explanation is imbalance between its synthesis and clearance (Afitska et al. 2019; Ghosh et al. 2017; Mehra et al. 2019). *SNCA* gene duplication, triplication, or mutation aggravates the accumulation of misfolded α-Syn (Chartier-Harlin et al. 2004; Conway et al. 1998; Ross et al. 2008). Moreover, the collapse of any pathways involved in α-Syn clearance and degradation results in the accumulation of α-Syn (Cuervo 2004; McNaught et al. 2002). For instance, disruption of the lysosomal degradation pathway promotes the formation of α-Syn inclusions in cells (Desplats et al. 2009).

Oxidative stress and mitochondrial dysfunction are also closely associated with PD pathophysiology (Hemmati-Dinarvand et al. 2019). Oxidative stress is a noxious condition induced by an imbalance between reactive oxygen species (ROS) production and detoxification. It ultimately leads to impairment of cellular function (Cenini et al. 2019). It is widely accepted that the mitochondrial respiratory chain is the primary source of ROS. Mitochondrial dysfunction can lead to a decline in energy production, generation of ROS, and induction of stress-induced apoptosis (Subramaniam and Chesselet 2013). On the one hand, mutations in some genes (such as *SNCA* and *LRRK2*) and mitochondrial DNA can disrupt the fission and fusion of mitochondria and contribute to mitochondrial dysfunction and the development of PD (Bose and Beal 2016; Nguyen et al. 2019). On the other hand, α-Syn fibrils can induce mitochondrial membrane depolarization, cytochrome C release, and mitochondrial fragmentation (Grassi et al. 2018). It has also been reported that mitochondrial oxidative stress leads to the accumulation of oxidized dopamine, resulting in lysosomal dysfunction and α-Syn accumulation (Burbulla et al. 2017).

It is hypothesized that neural hyperactivity at the early stages of PD can lead to pathophysiological degeneration (Jamwal and Kumar 2019). Glutamate is the most abundant excitatory neurotransmitter in the central nervous system, and alteration of glutamate homeostasis results in neurotoxic or excitotoxic events. One of the pathophysiological hallmarks of PD is the excessive release of glutamate into the SNpc, which activates ionotropic glutamate receptors and initiates the influx of Ca^2+^ ions (Himmelberg et al. 2018). This triggers a variety of destructive cascades. For example, visual stimulation increased neural activity and excitotoxicity in a *Drosophila* model of early-onset PD (Himmelberg et al. 2018). Furthermore, Yamada and coworkers found that elevated neuronal activity increased α-Syn release (Yamada and Iwatsubo 2018). These results indicate that neuronal excitotoxicity plays a vital role in the pathophysiology of PD.

PD diagnosis is based on the presence of bradykinesia and either resting tremor or rigidity and the absence of features from the history or examination, suggesting an alternative cause of parkinsonism (Reich and Savitt 2019). A panel of diseases or factors (such as PD, multiple system atrophy, and drug-induced Parkinsonism) can cause parkinsonism, a clinical syndrome including bradykinesia, cogwheel rigidity, resting tremor, slow shuffling gait, and imbalance (Hayes 2019). Because of its heterogeneous clinical manifestations, the diagnosis of PD is not easy. Therefore, it is essential to understand the molecular mechanisms of PD to promote diagnostic advances in the future.

## Tau Structure and Functions

Tau is encoded by the *MAPT* gene located on the long arm of chromosome 17 at 17q21 in humans. Alternative splicing of exons 2, 3, and 10 generates six Tau isoforms differing by one or two short inserts at the N-terminus (0 N, 1 N, and 2 N) and either three or four microtubule-binding repeat domains at the C-terminus (3R and 4R) (Goedert et al. 1989). Different Tau isoforms are expressed during development and in diseases. In the human fetal brain, only the shortest isoform of Tau is expressed; the mature brain expresses all six isoforms. Tau is mainly expressed in neurons but can also be detected in astrocytes and oligodendrocytes (Mietelska-Porowska et al. 2014). The N-terminal region of the protein contains a glycine-rich sequence, followed by two highly acidic regions and two proline-rich regions (P1 and P2); the remainder of the protein contains microtubule-binding domains and a short C-terminal region (Lee et al. 1988). As a member of the intrinsically disordered protein family, Tau shares many similarities with α-Syn. They are both abundant brain proteins with prion-like properties, as they can misfold, seed, and spread the misfolded conformation to typical monomeric forms of each protein (Vasili et al. 2019). Furthermore, an in vivo study demonstrated that Tau and α-Syn can accelerate each other’s aggregation, suggesting that Tau may be involved in the aggregation of α-Syn (Giasson 2003).

The physiological function of Tau is poorly understood. It was demonstrated to promote microtubule polymerization by interacting with the C-terminus of tubulin and driving tubulin assembly into microtubules, forming the cytoskeleton in neurons and defining neuronal morphology (Cleveland et al. 1977). Recent studies have demonstrated that Tau is concentrated on the labile domain of the axonal microtubules rather than on the stable domain, indicating that the role of Tau in the regulation of microtubule stability in the axon is not to stabilize axonal microtubules but to enable them to extend their labile domains (Baas and Qiang 2019). In addition to polymerizing microtubules and regulating their stability and mobility, Tau can regulate axonal transport. High concentrations of Tau bind to microtubules and differentially inhibit both dynein and kinesin functions, which are related to retrograde and anterograde transport of molecules in neurons, respectively (Dixit et al. 2008). However, Tau does not influence axonal transport within the physiological range (Tapia-Rojas et al. 2019), and studies have demonstrated that alterations in axonal transport can be observed only after Tau overexpression (Dubey et al. 2008). Thus, we can speculate that Tau influences axonal transport only under some pathological conditions.

In recent years, Tau has also been found in other subcellular structures and to exert different functions in these structures. First, nuclear Tau in its dephosphorylated state can protect DNA from damage, whereas Tau phosphorylation increases nuclear invagination and disrupts nucleocytoplasmic transport (Violet et al. 2014). One study demonstrated that hyperphosphorylated Tau interacts with components of the nuclear pore complex (NPC) to impair nucleocytoplasmic transport (Tripathi et al. 2019). Research on Tau-transgenic *Drosophila* showed that polyadenylated RNAs accumulate within and adjacent to Tau-induced nuclear envelope invaginations, leading to cell death (Cornelison et al. 2019). Paonessa et al. demonstrated that mutations in the *MAPT* gene result in microtubule-mediated deformation of the nucleus and disrupted nucleocytoplasmic transport (Paonessa et al. 2019). Second, under physiological conditions, low levels of Tau can be found at dendritic spines, where it regulates synaptic function in the dendritic/postsynaptic compartment (Frandemiche et al. 2014; Ittner et al. 2010). It is reported that Tau trans-locates to excitatory synapses in cultured mouse neurons and acute hippocampal slices, indicating that Tau might be involved in the regulation of synaptic plasticity (Frandemiche et al. 2014). Another study found that Tau participates in the postsynaptic targeting of the Src kinase Fyn. Additionally, knockout of Tau in mouse induces signal transduction disorder (Ittner et al. 2010).

Comprehensive investigations have revealed a role of Tau in physiology and pathology. Speculations on how Tau participate in or influence the pathophysiology of PD include the following: axonal transport dysfunction may contribute to the deposition of α-Syn and disable of excretion of metabolites (Dixit et al. 2008; Y. Wang and Mandelkow 2016); Tau translocation to the excitatory synapses may be involved in excitotoxicity in PD pathology (Frandemiche et al. 2014). Clarifying the association between Tau protein and PD is of interest, and additional research is warranted to explore possible mechanisms underlying PD.

## Association between *MAPT* Gene and PD

As the popularity of GWAS technology has grown, numerous studies have been performed to explore risk loci for PD (Edwards et al. 2010; International Parkinson’s Disease Genomics Consortium et al. 2017; International Parkinson’s Disease Genomics Consortium (IPDGC) et al. 2014). Thus far, at least 41 risk loci have been identified to be associated with PD (International Parkinson’s Disease Genomics Consortium et al. 2017), among which, *MAPT* is one of the most studied risk genes. Two extended haplotypes, H1 and H2, differ in orientation and do not recombine, covering the entire *MAPT* gene (Hutton 2000; Pastor et al. 2002; Stefansson et al. 2005). Variants in *MAPT* can increase the risk of PD and influence the progression and clinical manifestations of the disease. A full sequencing and haplotype analysis of *MAPT* in PD showed that the H1 haplotype is associated with an increased risk of PD but that the H2 haplotype has protective effects (Li et al. 2018). The pathologic effects of the H1 haplotype seem to vary in different populations. For example, the results of GWAS in Europe and America show a significant association (Mata et al. 2011; Refenes et al. 2009; Trotta et al. 2012; International Parkinson´s disease consortium 2011; Rhodes et al. 2010; Kalinderi et al. 2011). In contrast, the results of studies in Asia are not consistent (Chen et al. 2016; Li et al. 2018; Satake et al. 2009). Such a lack of consensus in investigations among different populations likely stems from the effects of population structure and population-specific environmental interactions, and the different results of studies in Asia may be caused by clinical heterogeneity and variable reliability in methodological issues. Hence, more studies are needed to explore the influences of *MAPT* on PD onset in the Asian population.

In addition to the influence on disease susceptibility, variations in *MAPT* also contribute to the clinical heterogeneity of PD. Some studies have discovered that *MAPT* is an independent risk factor for the development of cognitive impairment or dementia in PD patients (Setó-Salvia et al. 2011; Williams-Gray et al. 2009). Other studies found that the *MAPT* gene is also associated with the severity of motor symptoms (Huang et al. 2011; G. Wang et al. 2016). In a 5-year follow-up study, the *MAPT* H1/H1 genotype was proven to be an independent predictor of dementia risk in PD patients (Williams-Gray et al. 2009). Compta et al. reported that *MAPT* rs242557 is associated with high CSF Tau levels and low Aβ levels in PD patients (Compta et al. 2011a, b). Another gene-based and pathway-enrichment analysis suggested that *MAPT* is involved in the development of olfactory dysfunction, one of the most common nonmotor symptoms of PD, in older individuals (Dong et al. 2015). Genetic evidence suggests that the *MAPT* gene participates in disease onset and contributes to the diverse clinical manifestations of PD. Thus, identifying *MAPT* genotypes may help to identify groups at high risk for the development of PD and provide the possibility for early preventive measures. Furthermore, the implications of *MAPT* on PD indicate that Tau may have an essential role in the pathophysiology of PD.

## Post-mortem Observation of Tau in the PD Brain

In addition to α-Syn deposition, which is the characteristic pathology of PD, many studies have documented the occurrence of comorbid Tau in autopsy-confirmed PD brains (Table 1). Arima et al. found colocalization of Tau and α-Syn in LBs in PD as well as dementia with Lewy body (DLB) patients. They also classified the morphology of those inclusions into four types: (1) LBs with ring-shaped Tau immunoreactivity, (2) LBs surrounded by neurofibrillary tangles (NFTs), (3) α-Syn- and Tau-immunoreactive filamentous and granular masses, and (4) α-Syn- and Tau-immunoreactive dystrophic neurites (Arima et al. 1999). Another study excluded nonspecific antibody cross-reactivity by using a panel of monoclonal antibodies against Tau epitopes that span the entire length of the protein. The study showed colocalization of Tau and α-Syn in LBs, and most Tau-immunoreactive LBs were found in neurons vulnerable to NFTs, such as the locus coeruleus and basal nucleus of Meynert (Ishizawa et al. 2003). Galloway et al. found that an antibody specific for Tau does not react with LBs in the substantia nigra of isolated PD patients but can stain both the cortex and substantia nigra of dementia with Lewy bodies (DLB) patients (P G Galloway et al. 1988; Pamela G. Galloway et al. 1989). The discrepancy of these results may result from the variable reliability in the methodology applied.

**Table 1:** Post-mortem observation of Tau in the PD brain

Type of analysis	Cohorts	Findings	Citation (year)
Brain tissue	*N* = 7 -PD	1. Antibodies to tubulin, MAP1, MAP2, and NFT recognized LBs 2. Antibody specific to Tau was not incorporated into LBs	Galloway et al. (1988)
Brain tissue	*N* = 5 -DLB	Tau antibody stained inclusions in the cortex and substantia nigra	Galloway et al. (1989)
Brain tissue	*N* = 9 -PD (*n* = 2) -DLB (*n* = 7)	1. Both phosphorylation-dependent and independent Tau epitopes were present in LBs 2. Morphologies of colocalization of Tau and α-Syn can be classified into four types	Arima et al. (1999)
Brain tissue	*N* = 2 -DLB	1. α-Syn and Tau AT8 antibodies co-localized in LBs at the brain stem, cortex, and pale bodies 2. α-Syn and Tau aggregated into different filamentous components in the same inclusions	Arima et al. (2000)
Brain tissue	*N* = 24 -DLB/AD (*n* = 20) -AD (*n* = 4)	1. 80% of cases have Tau-immunoreactive LBs irrespective of the Braak stage 2. Tau immunostaining was present at the periphery of the LBs in most cases 3. The proportion of LBs with Tau immunoreactivity was most significant in neurons vulnerable to NFTs 4. The phospho-Tau antibody, TG3, detected more LBs than other Tau antibodies	Ishizawa et al. (2003)
Brain tissue	*N* = 56 -PDND (*n* = 27) -PDD (*n* = 29)	Cortical and striatal Aβ scores, Braak Tau stages, cortical Lewy body, Lewy neurite scores, and Lewy body densities, but not Braak α-Syn stages, were all significantly greater in PDD, with all the pathologies showing a significant positive correlation to each other	Compta et al. (2011a, b)
Brain tissue	*N* = 4 -PD (*n* = 2) -HD (*n* = 2)	1. Hyperphosphorylated Tau can be found in grafted tissue 16 years post- transplantation in PD patients 2. Hyperphosphorylated Tau can be found in grafted tissue 9 and 12 years post-transplantation in HD patients	Cisbani et al. (2017)
Brain tissue	*N* = 1 -PD	Immunohistochemical staining of graft tissue on 21 years post-transplantation PD patients demonstrated frequent neuronal perikaryal inclusions of phosphorylated α-Syn and Tau in the left graft only	Ornelas et al. (2020)

*PD* Parkinson’s disease, *AD* Alzheimer’s disease, *DLB* dementia with Lewy bodies, *PDND* Parkinson’s disease nondemented, *PDD* Parkinson’s disease-related dementia, *HD* Huntington's disease, *NFTs* nerve fiber tangles, *LBs* Lewy bodies

Cell replacement has been explored as a therapeutic strategy to treat PD. In addition to the postmortem observation that Tau is deposited in PD patients’ brains, Tau pathology has been observed in healthy grafts transplanted to PD patients. Cisbani and colleagues detected hyperphosphorylated Tau in grafted tissue of two PD patients at 18 months and 16 years posttransplantation, respectively. They also found Tau-positive inclusions, neurofibrillary tangles, and neuropil threads in patients who underwent autopsy at 16 years posttransplantation (Cisbani et al. 2017). Another case report showed frequent neuronal perikaryal inclusions positive for both phosphorylated α-Syn and Tau in the graft of a 70-year-old man who underwent cell transplantation 21 years prior (Ornelas et al. 2020). It is speculated that pathological α-Syn and Tau can spread from the host to the graft in a cell-to-cell manner. However, the transplantation process may also make the graft more susceptible to the spontaneous generation of pathology or factors within the host environment. These Tau pathologies observed in PD patients remind us that Tau may participate in PD pathophysiology by interacting with α-Syn or LBs. Overall, the different morphologies of Tau and α-Syn colocalization detected by immunohistochemistry technology indicate that more than one mechanism is involved (Arima et al. 1999, 2000).

## CSF Tau as a Biomarker for PD

PD can be subcategorized into different clinical subtypes based on etiology and clinical manifestations (Reich and Savitt 2019). Extensive investigations have attempted to develop biomarkers for the early and precise diagnosis of PD (Maass et al. 2019). Although many studies have shown that CSF α-Syn levels are lower in PD patients, a lack of association with the severity of disease and considerable overlap of values between control and diseased groups hamper the use of CSF α-Syn as a diagnostic marker (Delenclos et al. 2016; Maass et al. 2019). Hence, biomarkers that are more sensitive and specific are urgently needed to facilitate early and precise diagnosis and to track progression of the disease. Many investigations have explored the association between CSF Tau levels and the risk of developing PD or its clinical manifestations. Please see Table 2 for an overview of the literature.

**Table 2:** Clinical evidence supporting the role of Tau in PD pathophysiology

Type of sample	Cohorts	Findings	Citation (Year)
CSF	*N* = 70 -PDND (*n* = 20) -PDD (*n* = 20) -HC (*n* = 30)	CSF t-Tau and p-Tau levels: -higher in PDD than PDND and HC -associate with impaired memory and naming in PD	Compta et al. (2009)
CSF	*N* = 62 -PD (*n* = 32) -HC (*n* = 30)	1. Higher Tau and clusterin levels in PD versus HC 2. Higher Tau, Tau/Aβ_42_ and clusterin in patients suffering from PD less than 2 years versus those more than 2 years	Přikrylová Vranová et al. (2010)
CSF	*N* = 165 -PD (*n* = 109) -AD (*n* = 20) -HC (*n* = 36)	CSF t-Tau and p-Tau levels: -no different in HC versus PD without treatment	Alves et al. (2010)
CSF	*N* = 345 -PD (*n* = 49) -PD-CIND (*n* = 62) -PDD (*n* = 11) -AD (*n* = 49) -aMCI (*n* = 24) -HC (*n* = 150)	CSF t-Tau and p-Tau levels: -unchanged in PD-CIND and PDD CSF Aβ_42_ levels: -reduced in PD-CIND and PDD	Montine et al. (2010)
CSF	*N* = 121 -PDD (*n* = 21) -AD (*n* = 45) -DLB (*n* = 15) -HC (*n* = 40)	CSF Tau levels: -no difference between PDD and HC CSF Aβ levels: -lower in PDD versus HC	Bibl et al. (2010)
CSF	*N* = 56 -TDPD (*n* = 6) -NTPD (*n* = 6) -AD (*n* = 27) -HC (*n* = 17)	CSF t-Tau and Tau/Aβ_42_ levels: -significantly increased in both NTPD and AD compared with TDPD and HC groups	Jellinger (2012)
CSF	*N* = 22 -PD	CSF Aβ_42_, Aβ_42_/t-Tau, BDNF levels: -have significant associations with cognitive impairment in non-demented PD patients	Leverenz et al. (2011)
CSF	*N* = 181 -PD (*n* = 38) -DLB (*n* = 32) -AD (*n* = 48) -FTD (*n* = 31) -HC (*n* = 32)	1. Aβ_42_, t-Tau and p-Tau levels in PD patients were similar to controls 2. T-Tau/α-Syn and p-Tau/α-Syn showed the lowest values in PD patients	Parnetti et al. (2011)
CSF	PD (*n* = 48) -EDO-PD (*n* = 17) -TD-PD (*n* = 15) -NT-PD (*n* = 16) AD (*n* = 18) HC (*n* = 19)	In PD patients: -Tau and Tau/Aβ42 levels higher in NT-PD versus the other groups -Tau levels have a close relationship with motor manifestation in NT-PD	Přikrylová Vranová et al. (2012)
CSF	*N* = 403 -PD	Cross-sectional analyses: -baseline CSF biomarker levels positively correlated with each other -baseline CSF p-Tau/t-Tau and Aβ42 have borderline effects on the time to reach the endpoint Longitudinal analyses: -t-Tau and t-Tau/Aβ42 change rate are correlated with UPDRS total, or motor scores change rate	The Parkinson Study Group DATATOP Investigators et al. (2013)
CSF	*N* = 69 -PD (*n* = 44) -HC (*n* = 25)	1. o/t-α-Syn and Aβ_42_/t-Tau ratio significantly contributing to the discrimination of PD from HC 2. Patients with low CSF Aβ_42_ level are more prone to develop cognitive decline	Parnetti et al. (2014)
CSF	*N* = 403 -PD	CSF p-Tau and p-Tau/Aβ42 levels: -predict cognitive decline in PD since start treatment	Liu et al. (2015)
CSF	*N* = 390 -PD	Combination of age, non-motor assessments, DAT imaging, and CSF Aβ42/t-Tau ratio can predict the occurrence of cognitive impairment in PD patients during 2-years follow-up study	Schrag et al. (2017)
CSF	*N* = 285 -PD (*n* = 173) -HC (*n* = 112)	12 years longitudinal study in PD: -Aβ42 increase in both groups -t-Tau and α-Syn levels remained stable -p-Tau increased marginally more in PD over time -p-Tau/t-Tau increased, and t-Tau/Aβ42 decreased slightly Across time points: -t-Tau, p-Tau, and α-Syn levels were significantly lower in PD versus HC	Mollenhauer et al. (2017)
CSF Neuroimaging	PD = 421 -mild motor predominant (*n* = 223) -intermediate (*n* = 146) -diffuse malignant (*n* = 52)	1. Diffuse malignant PD have lowest Aβ and Aβ/t-Tau levels in CSF 2. MRI morphometry showed more atrophy and disease-specific network in diffuse malignant PD	Fereshtehnejad et al. (2017)
CSF Plasma	*N* = 115 -PD (*n* = 51) -HC-CSF (*n* = 40) -HC-Plasma (*n* = 24)	1. CSF levels of α-Syn, Aβ42, and TNF-α were lower in patients than in controls 2. The t-Tau/α-Syn, p-Tau/α-Syn, t-Tau/Aβ42 + α-Syn, and p-Tau/Aβ42 + α-Syn ratios were higher in patients 3. P-Tau/α-Syn alone and also combined with TNF-α obtained the best AUC 4. IL-6 positively correlated with UPDRS scores	Delgado-Alvarado (2017)
CSF	cohort 1 -PD (*n* = 281) cohort 2 -PD (*n* = 40)	1. T-Tau/Aβ42, t-Tau/α-Syn, t-Tau/Aβ42 + α-Syn, Aβ42/t-Tau ratios are associated with dementia risk over a 3-year follow-up 2. T-Tau/α-Syn and t-Tau/Aβ42 + α-Syn ratios are associated with progression to dementia over a 41-month follow-up	Delgado-Alvarado et al. (2018)
CSF	*N* = 136 -DLB (*n* = 51) -PD (*n* = 53) -HC (*n* = 32)	CSF Tau and p-Tau levels: -higher Tau levels in DLB versus PDD -higher Tau levels in PDD versus PD -Tau levels no difference between PD and HC -both reflect severity of dementia in PDD and DLB CSF p-Tau/Tau levels: -lower in DLB versus PDD	Gmitterová (2018)
CSF	*N* = 68 -PD (*n* = 30) -CBS (*n* = 11) -CBD (*n* = 8) -HC (*n* = 19)	1. 24-OHC levels increased in PD or CBS patients 2. CSF 24-OHC, Tau and p-Tau levels in PD, CBS or CBD patients correlate with each other	Björkhem et al. (2018)
CSF	*N* = 230 -PDND (*n* = 120) -HC (*n* = 110)	1. P-Tau levels were significantly lower in the PD group and rose significantly during the 1-year follow-up time in the PD group 2. T-Tau levels were different between the two groups at all time points despite their non-significant longitudinal changes	Dolatshahi et al. (2018)
CSF	*N* = 557 -PD (*n* = 415) -HC (*n* = 142)	10-year follow-up study of sporadic PD -low levels of Aβ_42_ are associated with a higher risk of developing cognitive impairment earlier in the disease process	Lerche et al. (2019)
Neuroimaging	PD (*n* = 30) -PD-CN (*n* = 15) -PD-MCI (*n* = 15) HC (*n* = 49)	Patterns of cortical Tau and Aβ do not differ in three groups	Winer et al. (2018)
Neuroimaging	*N* = 17 -PD	No significant increase in Tau tangles occurred after a two-year follow-up of PD patients	Hansen et al. (2020)

CSF Tau levels are significantly associated with cognitive impairments in PD patients. Several meta-analyses have shown that PD patients with cognitive impairment have elevated total Tau (t-Tau) and phosphorylated Tau (p-Tau) and reduced Aβ_42_ levels compared with those without cognitive impairment (Buongiorno et al. 2011; W. T. Hu et al. 2010; X. Hu et al. 2017). Leverenz et al. observed a significant association between CSF Aβ_42_, Aβ_42_/t-Tau, and BDNF levels and cognitive impairment in PD patients without dementia (Leverenz et al. 2011). Further studies showed that CSF p-Tau and p-Tau/Aβ_42_ levels could predict cognitive decline in PD patients (Liu et al. 2015; Schrag et al. 2017). Some studies have also suggested that the lifetime prevalence of cognitive impairment in PD is 80% (Aarsland et al. 2003; Buter 2008; Hely et al. 2008), making it very important to recognize PD patients who are more likely to develop cognitive impairment. Furthermore, CSF Tau levels may help in the differentiation of tremor-dominant PD (TDPD) and nontremor-dominant PD (NTPD). Indeed, it has been reported that t-Tau and Tau/Aβ_42_ levels are higher in NTPD patients than in TDPD patients and that Tau levels are closely related to motor symptoms in NTPD (Jellinger 2012; Přikrylová Vranová et al. 2012). Thus, CSF Tau levels may help in the diagnosis of PD subtypes and lay the foundation for developing personalized therapeutic strategies.

CSF Tau levels can also be used as a biomarker to track the progression of PD. In a longitudinal study of PD patients, the change rates of t-Tau and t-Tau/Aβ_42_ correlated with the Unified Parkinson Disease Rating Scale (UPDRS) total or motor score change rate (The Parkinson Study Group DATATOP Investigators et al. 2013). Another longitudinal study detected increased p-Tau and p-Tau/t-Tau and decreased t-Tau/Aβ_42_ in the CSF of PD patients in 12 years (Mollenhauer et al. 2017). Dolatshahi et al. found that p-Tau levels in CSF were low and rose significantly during the 1-year follow-up in the PD group (Dolatshahi et al. 2018). As CSF Tau has become an essential index for diagnosis of AD and other tauopathologies (Blennow and Zetterberg 2018; Takashima et al. 2019), clinical realities, such as manifestations, should be considered in differential diagnosis. However, its potential as a predicting factor of the course development and an indicator in PD’s typology should not be ignored.

## Experimental Evidence Supporting the Role of Tau in PD Pathophysiology

Various studies have employed transgenic PD mouse lines in which Tau is manipulated, reduced, or eliminated to explore the potential molecular pathways through which Tau may contribute to the pathophysiology of PD (Beauchamp 2018; Lei et al. 2012; Singh et al. 2019; Wills et al. 2011). Nevertheless, no consistent conclusion can be drawn thus far. The differences in the results of those studies may be due to intermodel variability. Some studies have been performed to explore changes in Tau protein in PD models. Wills and coworkers found increased hyperphosphorylated Tau in the striatum of adult A53T α-synuclein transgenic mice that colocalized with α-Syn, which was aggregated and accumulated in inclusion bodies (Wills et al. 2011). Further studies have shown that overexpression or mutation of α-Syn may increase the phosphorylation of Tau by promoting expression of GSK-3β, a primary kinase known to phosphorylate Tau at multiple sites (Ga and Adamczyk 2014; Kalinderi et al. 2011). Bardai et al. reported that the leucine-rich repeat kinase 2 (LRRK2) protein and mutations in the gene encoding it are the most common causes of familial PD and promote Tau neurotoxicity through dysregulation of actin and mitochondrial dynamics (Bardai et al. 2018). These studies suggest that Tau and α-Syn can promote each other’s pathological changes to form a vicious cycle, ultimately promoting the occurrence and development of PD.

Many in vitro studies have demonstrated that Tau and α-Syn can interact and promote aggregation of the other (Giasson 2003). Dasari and colleagues found that Tau interacts with the C-terminus of α-Syn and promotes the formation of toxic aggregations with distinct molecular conformations (Dasari et al. 2019). However, the results of in vivo studies remain controversial. Some research indicates that Tau is detrimental during the development of PD. Clinton and coworkers generated a mouse model expressing both α-Syn, Tau, and Aβ by mating 3xTg-AD mice (Tg(APPSwe, TauP301L)1Lfa) with A53T α-Synuclein transgenic mice and found that coexpression of the three proteins accelerated cognitive decline (Clinton et al. 2010). Singh et al. found aberrant localization of Tau to postsynaptic spines, which contributed to postsynaptic deficits and cognitive impairment in TgA53T mice. Furthermore, removal of endogenous Tau in A53T α-Synuclein transgenic mice ameliorated postsynaptic deficits and cognitive dysfunction (Singh et al. 2019; Teravskis et al. 2018). These results suggest that reducing Tau in the PD brain may break the vicious cycle of the two proteins and alleviate the symptoms of PD. Regardless, Morris and colleague reduced Tau levels in two kinds of PD models and found that this reduction failed to prevent motor deficits (Morris et al. 2011).

It has been reported that Tau-knockout mice develop motor deficits and cognitive impairment; thus, they have been used as an age-dependent model of parkinsonism in some studies. One study showed that knockout of Tau induced the accumulation of iron in dopaminergic neurons in the substantia nigra by impairing APP-mediated iron export. The authors found that Tau-knockout mice developed Parkinsonism and dementia (Lei et al. 2012). Leah C et al. reported olfactory and motor deficits in 7- and 15-month-old Tau-KO mice. They also found accumulation of α-Syn and autophagic impairment in the olfactory bulb, striatum, and substantia nigra at 7 and 15 months of age, respectively (Beauchamp 2018). The movement disorders caused by Tau deletion may be due to the loss of its normal function. These observations suggest that more caution should be taken when targeting Tau for the treatment of PD.

## Conclusions

Pathological and genetic evidence suggests that Tau plays an essential role in the pathogenesis of PD. However, the underlying molecular mechanisms remain unclear. Although a great deal of progress has been made in the field of PD, further studies are needed to develop methods for early and precise diagnosis as well as more efficient therapies. CSF Tau levels can help in PD diagnosis and monitoring disease progression. Moreover, crosstalk of Tau and α-Syn can induce loss of physiological function and axonal transport dysfunction, ultimately inducing the deposition of toxic fibrils and cell death. Overall, understanding the mechanisms by which Tau contributes to the pathophysiology of PD will be helpful for the treatment of PD in the future.

## Abbreviations

PD: Parkinson's disease
AD: Alzheimer's disease
PDND: Parkinson’s disease nondemented
PDD: Parkinson’s disease-related dementia
HC: Healthy control
PD-CIND: PD and cognitive impairment, not dementia
aMCI: Amnestic mild cognitive impairment
DLB: Dementia with Lewy bodies
TDPD: Tremor-dominant Parkinson’s disease
NTPD: Non-tremor-dominant Parkinson’s disease
FTD: Frontotemporal dementia
EDO-PD: Early disease onset Parkinson’s disease
CBS: Corticalbasal syndrome
CBD: Corticalbasal disease
PD-CN: Parkinson’s disease cognitively normal
PD-MCI: Parkinson’s disease with mild cognitive impairment
24-OHC: 24S-Hydroxycholesterol
UPDRS: Unified Parkinson Disease Rating Scale

## References

[CR1] Aarsland D, Andersen K, Larsen JP, Lolk A, Kragh-Sørensen P (2003). Prevalence and characteristics of dementia in Parkinson disease. Arch Neurol.

[CR2] Afitska K, Fucikova A, Shvadchak VV, Yushchenko DA (2019). α-Synuclein aggregation at low concentrations. Biochimica et Biophysica Acta (BBA) Proteins and Proteomics.

[CR3] Alves G, Bronnick K, Aarsland D, Blennow K, Zetterberg H, Ballard C (2010). CSF amyloid- and tau proteins, and cognitive performance, in early and untreated Parkinson’s Disease: The Norwegian ParkWest study. J Neurol Neurosurg Psychiatry.

[CR4] Arima K, Hirai S, Sunohara N, Aoto K, Izumiyama Y, Ueda K (1999). Cellular co-localization of phosphorylated tau- and NACPra-synuclein-epitopes in Lewy bodies in sporadic Parkinson’s disease and in dementia with Lewy bodies. Brain Res.

[CR5] Arima K, Mizutani T, Alim MA, Tonozuka-Uehara H, Izumiyama Y, Hirai S, Uéda K (2000). NACP/α-synuclein and tau constitute two distinctive subsets of filaments in the same neuronal inclusions in brains from a family of parkinsonism and dementia with Lewy bodies: Double-immunolabeling fluorescence and electron microscopic studies. Acta Neuropathol.

[CR6] Ascherio A, Schwarzschild MA (2016). The epidemiology of Parkinson’s disease: Risk factors and prevention. The Lancet Neurology.

[CR7] Baas PW, Qiang L (2019). Tau: It’s Not What You Think. Trends Cell Biol.

[CR8] Balestrino R, Schapira AHV (2020). Parkinson disease. Eur J Neurol.

[CR9] Bardai FH, Ordonez DG, Bailey RM, Hamm M, Lewis J, Feany MB (2018). Lrrk promotes tau neurotoxicity through dysregulation of actin and mitochondrial dynamics. PLoS Biol.

[CR10] Beauchamp LC (2018). Ablation of tau causes an olfactory deficit in a murine model of Parkinson’s disease. Acta Neuropathol Commun.

[CR11] Bibl M, Esselmann H, Lewczuk P, Trenkwalder C, Otto M, Kornhuber J (2010). Combined analysis of CSF Tau, A β 42, A β 1–42% and A β 1–40 ox % in Alzheimer’s disease, dementia with Lewy bodies and Parkinson’s disease dementia. Int J Alzheimers Dis.

[CR12] Björkhem I, Patra K, Boxer AL, Svenningsson P (2018). 24S-Hydroxycholesterol correlates with Tau and is increased in cerebrospinal fluid in Parkinson’s disease and corticobasal syndrome. Front Neurol.

[CR13] Blennow K, Zetterberg H (2018). Biomarkers for Alzheimer’s disease: Current status and prospects for the future. J Intern Med.

[CR14] Bose A, Beal MF (2016). Mitochondrial dysfunction in Parkinson’s disease. J Neurochem.

[CR15] Braak H, Tredici KD, Rüb U, de Vos RAI, Jansen Steur ENH, Braak E (2003). Staging of brain pathology related to sporadic Parkinson’s disease. Neurobiol Aging.

[CR16] Bras JM, Singleton A (2009) Genetic susceptibility in Parkinson’s disease. Biochimica et Biophysica Acta (BBA) - Mol Basis Dis 1792(7):597–603. 10.1016/j.bbadis.2008.11.008 10.1016/j.bbadis.2008.11.008 PMC2768897 19063963

[CR17] Buongiorno M, Compta Y, Martí MJ (2011). Amyloid-β and τ biomarkers in Parkinson’s disease–dementia. J Neurol Sci.

[CR18] Burbulla LF, Song P, Mazzulli JR, Zampese E, Wong YC, Jeon S (2017). Dopamine oxidation mediates mitochondrial and lysosomal dysfunction in Parkinson’s disease. Science.

[CR19] Buter TC (2008). Dementia and survival in Parkinson disease. Neurology.

[CR20] Cenini G, Lloret A, Cascella R (2019). Oxidative stress in neurodegenerative diseases: From a mitochondrial point of view. Oxid Med Cell Longev.

[CR21] Chartier-Harlin MC, Kachergus J, Roumier C, Mouroux V, Douay X, Lincoln S (2004). α-synuclein locus duplication as a cause of familial Parkinson’s disease. Lancet.

[CR22] Chaudhuri KR, Healy DG, Schapira AHV (2006). Non-motor symptoms of Parkinson’s disease: Diagnosis and management. Lancet Neurol.

[CR23] Chen Y, Cao B, Ou R, Chen X, Zhao B, Wei Q (2016). Association analysis of the *GRN* rs5848 and *MAPT* rs242557 polymorphisms in Parkinson’s disease and multiple system atrophy: A large-scale population-based study and meta-analysis. Int J Neurosci.

[CR24] Cisbani G, Maxan A, Kordower JH, Planel E, Freeman TB, Cicchetti F (2017). Presence of tau pathology within foetal neural allografts in patients with Huntington’s and Parkinson’s disease. Brain.

[CR25] Cleveland DW, Hwo SY, Kirschner MW (1977). Purification of tau, a microtubule-associated protein that induces assembly of microtubules from purified tubulin. J Mol Biol.

[CR26] Clinton LK, Blurton-Jones M, Myczek K, Trojanowski JQ, LaFerla FM (2010). Synergistic interactions between A, Tau, and -synuclein: acceleration of neuropathology and cognitive decline. J Neurosci.

[CR27] Compta Y, Parkkinen L, O’Sullivan SS, Vandrovcova J, Holton JL, Collins C (2011). Lewy- and Alzheimer-type pathologies in Parkinson’s disease dementia: Which is more important?. Brain.

[CR28] Compta Y, Ezquerra M, Muñoz E, Tolosa E, Valldeoriola F, Rios J (2011). High cerebrospinal tau levels are associated with the rs242557 tau gene variant and low cerebrospinal β-amyloid in Parkinson disease. Neurosci Lett.

[CR29] Compta Y, Martí MJ, Ibarretxe-Bilbao N, Junqué C, Tolosa E(2009) Cerebrospinal tau, phospho-tau, and beta-amyloid and neuropsychological functions in Parkinson’s disease. Mov Disord 24(15):2203–2210. 10.1002/mds.22594 10.1002/mds.22594 19795497

[CR30] Conway KA, Harper JD, Lansbury PT (1998). Accelerated in vitro fibril formation by a mutant α-synuclein linked to early-onset Parkinson disease. Nat Med.

[CR31] Cornelison GL, Levy SA, Jenson T, Frost B (2019). Tau-induced nuclear envelope invagination causes a toxic accumulation of mRNA in *Drosophila*. Aging Cell.

[CR32] Cuervo AM (2004). Impaired degradation of mutant-synuclein by chaperone-mediated autophagy. Science.

[CR33] Dasari AKR, Kayed R, Wi S, Lim KH (2019). Tau interacts with the C-terminal region of α-Synuclein, promoting formation of toxic aggregates with distinct molecular conformations. Biochem.

[CR34] Delenclos M, Jones DR, McLean PJ, Uitti RJ (2016). Biomarkers in Parkinson’s disease: advances and strategies. Parkinsonism Relat Disord.

[CR35] Delgado-Alvarado M (2017). Tau/a-Synuclein ratio and inflammatory proteins in Parkinson’s disease: an exploratory study. Mov Disord.

[CR36] Delgado-Alvarado M, Dacosta-Aguayo R, Navalpotro-Gómez I, Gago B, Gorostidi A, Jiménez-Urbieta H (2018). Ratios of proteins in cerebrospinal fluid in Parkinson’s disease cognitive decline: Prospective study: CSF protein ratios and dementia in PD. Mov Disord.

[CR37] Deng H, Wang P, Jankovic J (2018). The genetics of Parkinson disease. Ageing Res Rev.

[CR38] Desplats P, Lee HJ, Bae EJ, Patrick C, Rockenstein E, Crews L (2009). Inclusion formation and neuronal cell death through neuron-to-neuron transmission of -synuclein. Proc Natl Acad Sci.

[CR39] Dixit R, Ross JL, Goldman YE, Holzbaur ELF (2008). Differential regulation of dynein and kinesin motor proteins by Tau. Science.

[CR40] Dolatshahi M, Pourmirbabaei S, Kamalian A, Ashraf-Ganjouei A, Yaseri M, Aarabi MH (2018). Longitudinal alterations of alpha-Synuclein, amyloid beta, total, and phosphorylated Tau in cerebrospinal fluid and correlations between their changes in Parkinson’s disease. Front Neurol.

[CR41] Dong J, Yang J, Tranah G, Franceschini N, Parimi N, Alkorta-Aranburu G (2015). Genome-wide meta-analysis on the sense of smell among US older adults. Medicine.

[CR42] Dubey M, Chaudhury P, Kabiru H, Shea TB (2008). Tau inhibits anterograde axonal transport and perturbs stability in growing axonal neurites in part by displacing kinesin cargo: Neurofilaments attenuate tau-mediated neurite instability. Cell Motil Cytoskelet.

[CR43] Edwards TL, Scott WK, Almonte C, Burt A, Powell EH, Beecham GW (2010). Genome-wide association study confirms SNPs in *SNCA* and the *MAPT* region as common risk factors for Parkinson disease. Ann Hum Genet.

[CR44] Fereshtehnejad S-M, Zeighami Y, Dagher A, Postuma RB (2017). Clinical criteria for subtyping Parkinson’s disease: Biomarkers and longitudinal progression. Brain.

[CR45] Frandemiche ML, Seranno SD, Rush T, Borel E, Elie A, Arnal I (2014). Activity-dependent Tau protein translocation to excitatory synapse is disrupted by exposure to amyloid-beta oligomers. J Neurosci.

[CR46] Ga M, Adamczyk A (2014). Extracellular a-Synuclein leads to microtubule destabilization via GSK-3b-dependent Tau phosphorylation in PC12 cells. PLoS ONE.

[CR47] Galloway PG, Grundke-Iqbal I, Iqbal K, Perry G (1988). Lewy bodies contain epitopes both shared and distinct from Alzheimer neurofibrillary tangles. J Neuropathol Exp Neurol.

[CR48] Galloway PG, Bergeron C, Perry G (1989). The presence of tau distinguishes Lewy bodies of diffuse Lewy body disease from those of idiopathic Parkinson disease. Neurosci Lett.

[CR49] Ghosh D, Mehra S, Sahay S, Singh PK, Maji SK (2017). α-synuclein aggregation and its modulation. Int J Biol Macromol.

[CR50] Giasson BI (2003). Initiation and synergistic fibrillization of Tau and alpha-synuclein. Science.

[CR51] Gmitterová K (2018). Cerebrospinal fluid markers analysis in the differential diagnosis of dementia with Lewy bodies and Parkinson’s disease dementia. Eur Arch Psychiatry Clin Neurosci.

[CR52] Goedert M, Spillantini MC, Rutherford D, Crowther RA (1989). Multiple lsoforms of human microtubule-associated protein Tau: Sequences and localization in neurofibrillah tangles of Alzheimer’s disease. Neuron.

[CR53] Grassi D, Howard S, Zhou M, Diaz-Perez N, Urban NT, Guerrero-Given D (2018). Identification of a highly neurotoxic α-synuclein species inducing mitochondrial damage and mitophagy in Parkinson’s disease. Proc Natl Acad Sci.

[CR54] Hacker ML, Turchan M, Heusinkveld LE, Currie AD, Millan SH, Molinari AL (2020). Deep brain stimulation in early-stage Parkinson’s disease: five year outcomes. Neurology.

[CR55] Hansen AK, Parbo P, Ismail R, Østergaard K, Brooks DJ, Borghammer P (2020). Tau rangles in Parkinson’s disease: A 2-year follow-up flortaucipir PET study. J Parkinsons Dis.

[CR56] Hayes MT (2019). Parkinson’s disease and Parkinsonism. Am J Med.

[CR57] Hely MA, Reid WGJ, Adena MA, Halliday GM, Morris JGL (2008). The Sydney multicenter study of Parkinson’s disease: The inevitability of dementia at 20 years: Twenty Year Sydney Parkinson’s Study. Mov Disord.

[CR58] Hemmati-Dinarvand M, Saedi S, Valilo M, Kalantary-Charvadeh A, Alizadeh Sani M, Kargar R (2019). Oxidative stress and Parkinson’s disease: Conflict of oxidant-antioxidant systems. Neurosci Lett.

[CR59] Himmelberg MM, West RJH, Elliott CJH, Wade AR (2018). Abnormal visual gain control and excitotoxicity in early-onset Parkinson’s disease *Drosophila* models. J Neurophysiol.

[CR60] Hu WT, Chen-Plotkin A, Arnold SE, Grossman M, Clark CM, Shaw LM (2010). Biomarker discovery for Alzheimer’s disease, frontotemporal lobar degeneration, and Parkinson’s disease. Acta Neuropathol.

[CR61] Hu X, Yang Y, Gong D (2017). Changes of cerebrospinal fluid Aβ42, t-tau, and p-tau in Parkinson’s disease patients with cognitive impairment relative to those with normal cognition: A meta-analysis. Neurol Sci.

[CR62] Huang Y, Rowe DB, Halliday GM (2011). Interaction between α-Synuclein and Tau genotypes and the progression of Parkinson’s disease. J Parkinsons Dis.

[CR63] Hutton M (2000) Molecular genetics of chromosome 17 Tauopathies 920:63–73. 10.1111/j.1749-6632.2000.tb06906.x 10.1111/j.1749-6632.2000.tb06906.x 11193178

[CR64] International Parkinson Disease Genomics Consortium, Nalls MA, Plagnol V, Hernandez DG, Sharma M, Sheerin UM et al (2011) Imputation of sequence variants for identification of genetic risks for Parkinson's disease: a meta-analysis of genome-wide association studies. Lancet 377(9766):641–649. 10.1016/S0140-6736(10)62345-8 10.1016/S0140-6736(10)62345-8 PMC3696507 21292315

[CR65] International Parkinson’s Disease Genomics Consortium, 23 and Me Research Team, Chang D, Nalls MA, Hallgrímsdóttir IB, Hunkapiller J et al (2017) A meta-analysis of genome-wide association studies identifies 17 new Parkinson’s disease risk loci. Nat Gen 49(10):1511–1516. 10.1038/ng.3955 10.1038/ng.3955 PMC5812477 28892059

[CR66] International Parkinson’s Disease Genomics Consortium (IPDGC), Parkinson’s Study Group (PSG) Parkinson’s Research: The Organized GENetics Initiative (PROGENI), 23andMe, GenePD, NeuroGenetics Research Consortium (NGRC), Hussman Institute of Human Genomics (HIHG) et al (2014) Large-scale meta-analysis of genome-wide association data identifies six new risk loci for Parkinson’s disease. Nat Gen 46(9):989–993. 10.1038/ng.3043 10.1038/ng.3043 PMC4146673 25064009

[CR67] Ishizawa T, Mattila P, Davies P, Wang D, Dickson DW (2003). Colocalization of Tau and alpha-Synuclein epitopes in Lewy bodies. J Neuropathol Exp Neurol.

[CR68] Ittner LM, Ke YD, Delerue F, Bi M, Gladbach A, van Eersel J (2010). Dendritic function of Tau mediates amyloid-β toxicity in Alzheimer’s disease mouse models. Cell.

[CR69] Jamwal S, Kumar P (2019). Insight into the emerging role of striatal neurotransmitters in the pathophysiology of Parkinson’s disease and Huntington’s disease: A review. Curr Neuropharmacol.

[CR70] Jellinger KA (2012). CSF biomarkers in different phenotypes of Parkinson disease. J Neural Trans.

[CR71] Kalinderi K, Fidani L, Katsarou Z, Clarimón J, Bostantjopoulou S, Kotsis A (2011). GSK3β polymorphisms, MAPT H1 haplotype and Parkinson’s disease in a Greek cohort. Neurobiol Aging.

[CR72] Kaur R, Mehan S, Singh S (2019). Understanding multifactorial architecture of Parkinson’s disease: pathophysiology to management. Neurol Sci.

[CR73] Krack P, Batir A, Van Blercom N, Chabardes S, Fraix V, Ardouin C (2003). Five-year follow-up of bilateral stimulation of the subthalamic nucleus in advanced Parkinson’s disease. N Engl J Med.

[CR74] Lee Gl, Cowan N, Kirschner M (1988). The primary structure and heterogeneity of Tau protein from mouse brain. Science.

[CR75] Lei P, Ayton S, Finkelstein DI, Spoerri L, Ciccotosto GD, Wright DK (2012). Tau deficiency induces parkinsonism with dementia by impairing APP-mediated iron export. Nat Med.

[CR76] Lerche S, Wurster I, Röben B, Machetanz G, Zimmermann M, Bernhard F (2019). Parkinson’s disease: Evolution of cognitive impairment and CSF Aβ _1–42_ profiles in a prospective longitudinal study. J Neurol Neurosurg Psychiatry.

[CR77] Leverenz JB, Stennis Watson G, Shofer J, Zabetian CP, Zhang J, Montine TJ (2011). Cerebrospinal fluid biomarkers and cognitive performance in non-demented patients with Parkinson’s disease. Parkinsonism Relat Disor.

[CR78] Li J, Ruskey JA, Arnulf I, Dauvilliers Y, Hu MTM, Högl B (2018). Full sequencing and haplotype analysis of *MAPT* in Parkinson’s disease and rapid eye movement sleep behavior disorder: *MAPT* in PD and RBD. Mov Disord.

[CR79] Liu C, Cholerton B, Shi M, Ginghina C, Cain KC, Auinger P, Zhang J (2015). CSF tau and tau/Aβ42 predict cognitive decline in Parkinson’s disease. Parkinsonism Relat Disor.

[CR80] Maass F, Schulz I, Lingor P, Mollenhauer B, Bähr M (2019). Cerebrospinal fluid biomarker for Parkinson’s disease: An overview. Mol Cell Neurosci.

[CR81] Mata IF, Yearout D, Alvarez V, Coto E, de Mena L, Ribacoba R (2011). Replication of MAPT and SNCA, but not PARK16-18, as susceptibility genes for Parkinson’s disease. Mov Disord.

[CR82] McNaught KSP, Björklund LM, Belizaire R, Isacson O, Jenner P, Olanow CW (2002) Proteasome inhibition causes nigral degeneration with inclusion bodies in rats: Neuroreport 13(11):1437–1441. 10.1097/00001756-200208070-00018 10.1097/00001756-200208070-00018 12167769

[CR83] Mehra S, Sahay S, Maji SK (2019) α-Synuclein misfolding and aggregation: Implications in Parkinson’s disease pathogenesis. Biochimica et Biophysica Acta (BBA) - Proteins Proteom 1867(10):890–908. 10.1016/j.bbapap.2019.03.001 10.1016/j.bbapap.2019.03.001 30853581

[CR84] Mietelska-Porowska A, Wasik U, Goras M, Filipek A, Niewiadomska G (2014). Tau protein modifications and interactions: their role in function and dysfunction. Int J Mol Sci.

[CR85] Mollenhauer B, Caspell-Garcia CJ, Coffey CS, Taylor P, Shaw LM, Trojanowski JQ (2017). Longitudinal CSF biomarkers in patients with early Parkinson disease and healthy controls. Neurol.

[CR86] Montine TJ, Shi M, Quinn JF, Peskind ER, Craft S, Ginghina C (2010). CSF Aβ _42_ and tau in Parkinson’s disease with cognitive impairment: CSF biomarkers for PD with CI or dementia. Mov Disord.

[CR87] Morris M, Koyama A, Masliah E, Mucke L (2011). Tau reduction does not prevent motor deficits in two mouse models of Parkinson’s disease. PLoS ONE.

[CR88] Nguyen M, Wong YC, Ysselstein D, Severino A, Krainc D (2019). Synaptic, mitochondrial, and lysosomal dysfunction in Parkinson’s disease. Trends Neurosci.

[CR89] Ornelas AS, Adler CH, Serrano GE, Curry JR, Shill HA, Kopyov O, Beach TG (2020). Co-Existence of tau and α-synuclein pathology in fetal graft tissue at autopsy: A case report. Parkinsonism Relat Disor.

[CR90] Paonessa F, Evans LD, Solanki R, Larrieu D, Wray S, Hardy J (2019). Microtubules deform the nuclear membrane and disrupt nucleocytoplasmic transport in tau-mediated frontotemporal dementia. Cell Rep.

[CR91] Parnetti L, Chiasserini D, Bellomo G, Giannandrea D, De Carlo C, Qureshi MM (2011). Cerebrospinal fluid Tau/α-synuclein ratio in Parkinson’s disease and degenerative dementias. Mov Disord.

[CR92] Parnetti L, Farotti L, Eusebi P, Chiasserini D, De Carlo C, Giannandrea D (2014). Differential role of CSF alpha-synuclein species, tau, and Aβ42 in Parkinson’s disease. Front Aging Neurosci.

[CR93] Pastor P, Ezquerra M, Tolosa E, Muñoz E, José Martí M, Valldeoriola F (2002). Further extension of the H1 haplotype associated with progressive supranuclear palsy: Extension of the H1 Haplotype Associated with PSP. Mov Disord.

[CR94] Přikrylová Vranová H, Mareš J, Hluštík P, Nevrlý M, Stejskal D, Zapletalová J (2012). Tau protein and beta-amyloid1-42 CSF levels in different phenotypes of Parkinson’s disease. J Neural Transm.

[CR95] Přikrylová Vranová H, Mareš J, Nevrlý M, Stejskal D, Zapletalová J, Hluštík P, Kaňovský P (2010). CSF markers of neurodegeneration in Parkinson’s disease. J Neural Transm.

[CR96] Refenes N, Bolbrinker J, Tagaris G, Orlacchio A, Drakoulis N, Kreutz R (2009). Role of the H1 haplotype of microtubule-associated protein tau (MAPT) gene in Greek patients with Parkinson’s disease. BMC Neurol.

[CR97] Reich SG, Savitt JM (2019). Parkinson’s disease. Med Clin North Am.

[CR98] Rhodes SL, Sinsheimer JS, Bordelon Y, Bronstein JM, Ritz B (2010). Replication of GWAS associations for GAK and MAPT in Parkinson’s disease: Replication of GAK and MAPT associations in PD. Ann Hum Genet.

[CR99] Ross OA, Braithwaite AT, Skipper LM, Kachergus J, Hulihan MM, Middleton FA (2008). Genomic investigation of α-synuclein multiplication and parkinsonism. Ann Neurol.

[CR100] Satake W, Nakabayashi Y, Mizuta I, Hirota Y, Ito C, Kubo M (2009). Genome-wide association study identifies common variants at four loci as genetic risk factors for Parkinson’s disease. Nat Genet.

[CR101] Schrag A, Siddiqui UF, Anastasiou Z, Weintraub D, Schott JM (2017). Clinical variables and biomarkers in prediction of cognitive impairment in patients with newly diagnosed Parkinson’s disease: A cohort study. Lancet Neurol.

[CR102] Setó-Salvia N, Clarimón J, Pagonabarraga J, Pascual-Sedano B, Campolongo A, Combarros O (2011). Dementia risk in Parkinson disease: Disentangling the role of MAPT haplotypes. Arch Neurol.

[CR103] Shi M, Kovac A, Korff A, Cook TJ, Ginghina C, Bullock KM (2016). CNS tau efflux via exosomes is likely increased in Parkinson’s disease but not in Alzheimer’s disease. Alzheimers Dement.

[CR104] Singh B, Covelo A, Martell-Martínez H, Nanclares C, Sherman MA, Okematti E (2019). Tau is required for progressive synaptic and memory deficits in a transgenic mouse model of α-synucleinopathy. Acta Neuropathol.

[CR105] Stefansson H, Helgason A, Thorleifsson G, Steinthorsdottir V, Masson G, Barnard J (2005). A common inversion under selection in Europeans. Nat Genet.

[CR106] Subramaniam SR, Chesselet M-F (2013). Mitochondrial dysfunction and oxidative stress in Parkinson’s disease. Prog Neurobiol.

[CR107] Sveinbjornsdottir S (2016). The clinical symptoms of Parkinson’s disease. J Neurochem.

[CR108] Takashima A, Wolozin B, Buee L (Eds.) (2019) Tau Biology (Vol. 1184) Singapore: Springer Singapore. 10.1007/978-981-32-9358-8

[CR109] Tapia-Rojas C, Cabezas-Opazo F, Deaton CA, Vergara EH, Johnson GVW, Quintanilla RA (2019). It’s all about tau. Prog Neurobiol.

[CR110] Teravskis PJ, Covelo A, Miller EC, Singh B, Martell-Martínez HA, Benneyworth MA (2018). A53T mutant alpha-synuclein induces Tau-dependent postsynaptic impairment independently of neurodegenerative changes. J Neurosci.

[CR111] The Parkinson Study Group DATATOP Investigators, Zhang J, Mattison HA, Liu C, Ginghina, C, Auinger, P et al (2013) Longitudinal assessment of tau and amyloid beta in cerebrospinal fluid of Parkinson disease Acta Neuropathol 126(5):671–682. 10.1007/s00401-013-1121-x 10.1007/s00401-013-1121-x PMC3796193 23644819

[CR112] Tripathi T, Prakash J, Shav-Tal Y (2019). Phospho-Tau impairs nuclear-cytoplasmic transport. ACS Chemical Neuroscience.

[CR113] Trotta L, Guella I, Soldà G, Sironi F, Tesei S, Canesi M (2012). SNCA and MAPT genes: Independent and joint effects in Parkinson disease in the Italian population. Parkinsonism Relat Disor.

[CR114] Vasili E, Dominguez-Meijide A, Outeiro TF (2019). Spreading of α-Synuclein and Tau: A systematic comparison of the mechanisms involved. Front Mol Biosci.

[CR115] Violet M, Delattre L, Tardivel M, Sultan A, Chauderlier A, Caillierez R (2014). A major role for Tau in neuronal DNA and RNA protection in vivo under physiological and hyperthermic conditions. Front Cell Neurosci.

[CR116] Wakabayashi K, Tanji K, Odagiri S, Miki Y, Mori F, Takahashi H (2013). The Lewy body in Parkinson’s disease and related neurodegenerative disorders. Mol Neurobiol.

[CR117] Wang G, Huang Y, Chen W, Chen S, Wang Y, Xiao Q (2016). Variants in the SNCA gene associate with motor progression while variants in the MAPT gene associate with the severity of Parkinson’s disease. Parkinsonism Relat Disor.

[CR118] Wang Y, Mandelkow E (2016). Tau in physiology and pathology. Nat Rev Neurosci.

[CR119] Williams-Gray CH, Evans JR, Goris A, Foltynie T, Ban M, Robbins TW (2009). The distinct cognitive syndromes of Parkinson’s disease: 5 year follow-up of the CamPaIGN cohort. Brain.

[CR120] Wills J, Credle J, Haggerty T, Lee J-H, Oaks AW, Sidhu A (2011). Tauopathic changes in the striatum of A53T α-Synuclein mutant mouse model of Parkinson’s disease. PLoS ONE.

[CR121] Winer JR, Maass A, Pressman P, Stiver J, Schonhaut DR, Baker SL (2018). Associations between Tau, β-amyloid, and cognition in Parkinson disease. JAMA Neurology.

[CR122] Yamada K, Iwatsubo T (2018). Extracellular α-synuclein levels are regulated by neuronal activity. Mol Neurodegener.

